# Toads on Lava: Spatial Ecology and Habitat Use of Invasive Cane Toads (*Rhinella marina*) in Hawai’i

**DOI:** 10.1371/journal.pone.0151700

**Published:** 2016-03-30

**Authors:** Georgia Ward-Fear, Matthew J. Greenlees, Richard Shine

**Affiliations:** School of Life and Environmental Sciences, Heydon-Laurence Building A08, University of Sydney, Sydney, New South Wales, Australia; Department of Agriculture and Water Resources, AUSTRALIA

## Abstract

Most ecological research on cane toads (*Rhinella marina*) has focused on invasive populations in Australia, ignoring other areas where toads have been introduced. We radio-tracked and spool-tracked 40 toads, from four populations on the island of Hawai’i. Toads moved extensively at night (mean 116 m, from spool-tracking) but returned to the same or a nearby retreat-site each day (from radio-tracking, mean distance between successive retreat sites 11 m; 0 m for 70% of records). Males followed straighter paths during nocturnal movements than did females. Because moist sites are scarce on the highly porous lava substrate, Hawai’ian toads depend on anthropogenic disturbance for shelter (e.g. beneath buildings), foraging (e.g. suburban lawns, golf courses) and breeding (artificial ponds). Foraging sites are further concentrated by a scarcity of flying insects (negating artificial lights as prey-attractors). Habitat use of toads shifted with time (at night, toads selected areas with less bare ground, canopy, understory and leaf-litter), and differed between sexes (females foraged in areas of bare ground with dense understory vegetation). Cane toads in Hawai’i thrive in scattered moist patches within a severely arid matrix, despite a scarcity of flying insects, testifying to the species’ ability to exploit anthropogenic disturbance.

## Introduction

Even for intensively-studied “model organisms”, field research often has focused primarily on populations within a small part of the species’ overall range: for example, where the organisms are easy to study, or where they inflict significant economic, medical or environmental damage [1–3]. Thus, many introduced species have attracted intensive study within their invaded range (where they are a problem) but not their native range [4–6]. However, to fully understand the biology of such a taxon, we need to study it in a variety of places, including those where it is not a high-profile pest.

The international diaspora of the cane toad (*Rhinella marina*) offers a clear example of geographically heterogeneous research effort. Although translocated from the Americas to >40 countries worldwide [5,7], the species has attracted minimal research (and may have had little environmental impact) in most of those recipient areas. In contrast, cane toads have attracted intensive research in Australia, where they have inflicted major ecological carnage by fatally poisoning apex predators [8,9]. Our detailed understanding of the ecology of the cane toad (arguably, as well-known as any anuran species) rests almost entirely on research conducted within Australia [7].

Cane toads were introduced to O’ahu in 1932 (from Puerto Rico) to control beetle pests in commercial sugar-cane plantations [7, 10]. More than 100,000 offspring of the original 150 founders were collected and released on the Hawai’ian Islands over the next few years [7]. Those introductions involved both the wet side and dry side of Hawai’i, because artificial irrigation allowed sugar cane to be grown even in low-rainfall areas [11]. Cane toads are now so common that they have their own name (“poloka”) in the Hawai’ian language [12].

The 101 toads that founded the Australian population in 1935 were collected in Hawai’i, from a population established there in 1932 (from South America, via Puerto Rico [7,13]). Thus, the ecology of the Hawai’ian population of cane toads may shed light both on the progenitors of the Australian toads, and on the influence of 80 years of Hawai’ian conditions on toad ecology. Such information can, for example, clarify the way that toad ecology is affected by xeric conditions (as occur in northwestern Australia, and on the western side of each Hawai’ian island), as opposed to mesic conditions (as occur in northeastern Australia, and on the eastern side of each Hawai’ian island). Also, studies from Hawai’i can help to test hypotheses based on Australian studies. For example, high dispersal rates of toads in northwestern Australia have been attributed to evolutionary pressures exerted by the invasion process [14–16]; and if so, we would not expect to see equivalently high dispersal rates in toads lacking a history of sustained range expansion. More broadly, data on Hawai’ian toad movements can test the assumption that low dispersal rates of toads in Australia are the ancestral condition, and that high dispersal rates are derived [15]. Thus, we radio-tracked and spool-tracked adult cane toads on the island of Hawai’i in June 2015 to quantify patterns of movement and habitat use of free-ranging toads from a range of sites.

## Materials and Methods

### Study species

Native to a broad area extending from Mexico through Brazil, cane toads (*Rhinella* [*Bufo*] *marina*) are one of the largest bufonid anurans [17]. Adult cane toads are almost exclusively nocturnal, spending the day in sheltered retreat-sites and emerging at night to feed, rehydrate, and breed [7,18].

### Abiotic and biotic constraints in Hawai’i

**Fig 1:**
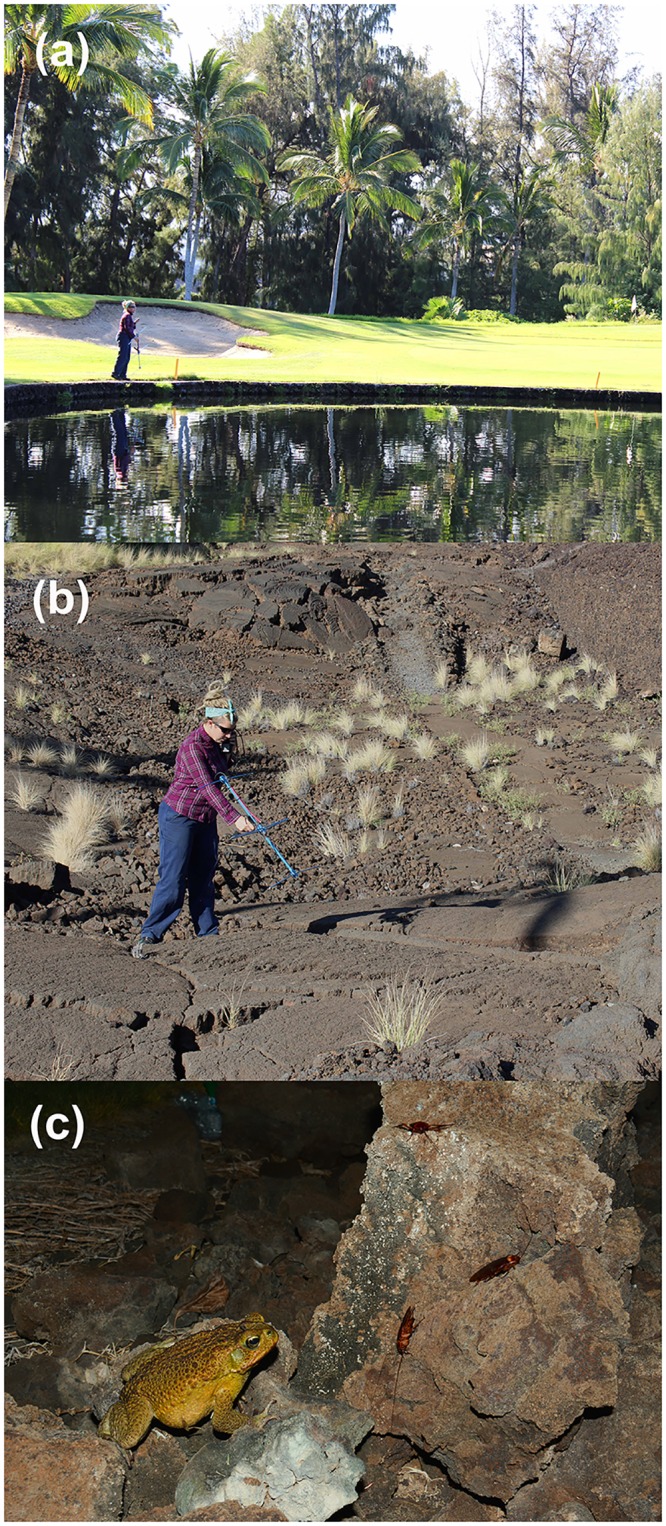
General habitat use by toads on the island of Hawai’i. On the leeward (dry) side of Hawai’i, dense populations of cane toads were found only in artificially irrigated areas like this golf course (a); but toads tracked at these sites often used crevices in adjacent lava flows as diurnal retreat sites (b). Observations and dissections suggest that cockroaches were the most important prey type for all toad populations that we studied (c). Photographs by M. Greenlees (a,b) and G. Clarke (c).

The distinctive landforms and climate of Hawai’i impose strong restrictions on anurans (Fig 1). Due to the islands’ volcanic origin, the substrate is composed of highly porous lava; thus, damp retreat-sites are scarce. Rainfall is high and relatively aseasonal on the eastern windward (wet) side of the major islands, but extensive rain-shadows create severely arid conditions on the western leeward (dry) side [19]. Even on the wet side of each island, natural permanent waterbodies are rare. Allied to that scarcity, the depauperate native insect fauna of Hawai’i offers limited feeding opportunities for a generalist insectivore such as the cane toad. For example, artificial lights attract swarms of flying insects (at least seasonally) in many parts of the tropics, but not in Hawai’i (see below for quantification of this pattern). However, anthropogenically modified habitat provides both water and insect food. On the wet side of the island of Hawai’i, frequent rainfall allows toads to move extensively through the landscape to exploit favorable (human-subsidized) resource patches; but on the dry side, toads are primarily restricted to artificially-watered habitats such as golf courses.

### Distributions

In the course of collecting toads for other studies, we searched at night for anurans in suitable (moist, vegetated) sites on both the windward and leeward sides of O’ahu, Maui and Hawai’i. We did not quantify abundances, but made general notes about numbers encountered.

### Study areas

We studied toads at two sites on the eastern windward (wet,) side of the island of Hawai’i, and two sites on the western leeward (dry,) side, as follows:

1. Dry side 1: Mauna Kea golf course 50 km north of Kailua-Kona. The surrounding landscape is highly arid (mean annual rainfall from 1981 to 2010 = 467 mm [20]), dominated by lava flows. In contrast, the golf course is lush lawn, watered daily by automatic sprinklers. Toads at this site (3 males, 7 females) sheltered by day predominantly in artificial rock walls, and emerged onto the golf course to feed at night.
2. Dry side 2: 15 km south of Dry side 1, Waikoloa Beach Resort resembles Dry side 1 in most respects. Resort and shopping complexes are surrounded by golf courses with well-watered lawns and artificial waterbodies, bounded in turn by a bare landscape of lava flows. Toads at this site (5 males, 5 females) utilized artificial refugia, but also sheltered in crevices within lava flows.
3. Wet side 1: 23 km south of Hilo, we captured toads on open grassy areas around a large house we used as a research base (“Toad Hall”). The windward side of the island historically was dominated by lush forests (mean annual rainfall from 1981 to 2010 = 3219 mm [20]), but now also supports agricultural and urban development. By day, the toads (5 males, 5 females) generally sheltered in crevices and burrows among lava rubble in a mixed fruit and vegetable market-garden, and in planted gardens. Toads emerged at night to forage on an extensive patch of mowed lawn and throughout the market-garden.
4. Wet side 2: immediately adjacent to the campus of the University of Hawai’i at Hilo (19°42’10.42”N, 155°04’48.51”W), our toads (2 males, 8 females) sheltered beneath buildings and inside drains by day, and emerged at night onto grassy areas. One large (560 g) female toad was found feeding on cat-food (and snails attracted to that cat-food) that had been placed out near a building.

### Insect abundances

We scored the number of flying insects within a 1-m radius of artificial lights (external wall lights) during the two hours following dusk. To do so, we marked a 1-m quadrat on the vertical wall (centered on the light), then left for 2 min; upon our return we counted insects within the quadrat. Counts were completed within 60 seconds, and only included insects in contact with the wall. We surveyed 24 independent lights on the dry side of Hawai’i and 32 lights on the wet side; each light was visited only once during the study, within a period of five days. For comparison, we took equivalent data in tropical Australia (Middle Point, 12°34’42.12”S, 131°18’51.98”E), at a site where much previous research has been conducted on cane toads [21]. Fifteen lights were surveyed in the dry season (May to October) and 17 in the wet season (November to April).

### Radio-tracking

We captured toads at night, and measured and weighed them. Sex was determined from morphology (skin rugosity and color [22]) and the male-specific “release call” [23]. We attached radio-transmitters (Holohil PD-2, ~3.5 g, <5% of toad mass) to cotton twine waist-belts. Toads were released at their point of capture, either the same night or the following evening. We located each animal daily (in its diurnal retreat-site), at which time we took a GPS reading, a temperature reading of its retreat using an infra-red thermometer (Digitech, QM7221, accuracy ±1%) and scored habitat attributes of the area within a 1-m radius of the shelter-site (proportion of quadrat substrate consisting of low [<10 cm] vegetation, bare ground, leaf litter, mean height of understory vegetation, mean depth of leaf litter, % canopy cover, canopy height). We repeated these measurements at a site 5 m from the shelter site, in a randomly-selected direction (by following the point of a pencil which had been laid on a flat surface and ‘spun’), to quantify habitat availability as well as use. Toads were located, and habitat data recorded, every day for five days. Toads were then recaptured, and the radio-transmitters were removed before the animals were released.

### Spool-tracking

To clarify toad movements at night, we attached a small (4 cm x 1 cm) nylon spool (2.5 g) to the waistbelt holding the transmitter, and tied the free end to a nearby stick. Each telemetered toad was spool-tracked on a single night only (the last night of tracking). We were unable to spool-track toads at Dry side 1 (for logistical reasons). To maintain equal sample sizes for dry-side and wet-side sites overall, we spool-tracked an additional nine non-telemetered toads (5 males, 4 females) at Dry side 2. We followed the spool track the following day to score the total distance moved by each toad, its net displacement over the same period, and habitat attributes (as above, in a 1-m^2^ quadrat) at 10-m intervals along the spool. To quantify habitat availability, we scored the same attributes at nearby random sites (5 m from the spool line, alternating on the right and left sides).

### Statistical analyses

With the program JMP 11.0 (SAS Institute, Cary, NC), we used ANOVA to examine the effects of toad sex and site on distances between successive diurnal retreat-sites, habitat characteristics of sites used by toads by day and by night (as well as nearby available habitats), and net displacements relative to total distance travelled. Preliminary analyses revealed no significant effects of toad body size on movement or habitat parameters (all p > 0.20), so this variable was deleted from the analyses. Similarly, we looked for an effect of day #, to see if toads tended to behave differently (e.g., move further) on the first day post-release (in response to handling stress) than on later days. None of our dependent variables was significantly affected by day #, even if we included the single large movement made by one toad on the day it was released (p = 0.09 with that data point included; p = 0.20 without it). We retained site as a factor in all analyses. To control for repeated measures (5 nights and 5 days) per toad, we calculated an average value for each toad (over the entire tracking period) for each variable (separately for data taken by day versus at night), and included toad ID as a random factor in the ANOVAs.

### Ethics statement

Our protocol was approved by the University of Sydney’s Animal Ethics Committee (Permit number 2013/6034). Methods were non-invasive and the duration of interference (radio-tracking) was short. Nevertheless, all care was taken to minimize any distress experienced by the animals. The individual shown in the photograph in this manuscript has given written informed consent (as outlined in PLoS consent form) to publish these case details.

## Results

### Distribution and body sizes

Cane toads were abundant on both windward (wet) and leeward (dry) sides of the three islands we sampled (Table 1). The only other commonly-encountered species was the coqui frog (*Eleutherodactylus coqui*) that was abundant in suburban gardens at some windward-side locations. Cane toads were by far the most widely distributed anuran, especially on the leeward sides of each island (Table 1). In our sample of 40 adult toads radio-tracked on the island of Hawai’i in June 2015, females averaged larger than males (mean snout-urostyle length [SUL] 106.7 mm [SE = 2.39] vs. 99.4 mm [SE = 2.22]; mean mass 163.8 g [SE = 20.67] vs. 125.0 g [SE = 8.97]). Mean SUL per site ranged from 99 to 107 mm, and mass from 113 to 176 g.

**Table 1:** Occurrence and relative abundance of anurans encountered during nocturnal surveys on windward and leeward sides of O’ahu, Hawai’i (“the Big Island”) and Maui.

Island	Side of island	Cane Toads *Rhinella marina*	Common Coqui *Eleutherodactylus coqui*	Greenhouse frog *E*. *planirostris*	Bullfrog *Lithobates catesbianus*	Other species detected*
O’ahu	Leeward	abundant	not detected	not detected	not detected	
O’ahu	Windward	abundant	not detected	common	abundant	*Dendrobates auratus*, *Glandirana rugosa*
Hawai’i	Leeward	abundant	scarce	scarce	not detected	
Hawai’i	Windward	abundant	abundant	scarce	abundant	
Maui	Leeward	abundant	scarce	common	not detected	
Maui	Windward	abundant	common	common	scarce	*Glandirana rugosa*

* other species detected were all highly localized and scarce. *Dendrobates auratus* (green and black poison dart frog), *Glandirana rugosa* (Japanese wrinkled frog).

### Insect abundance

During our surveys of artificial lights at night on Hawai’i (N = 56 lights with a single count each; dry side N = 24, wet side N = 32), we recorded an average of 1.2 (SE = 1.1, range 0 to 4) insects within 1 m of the light. Insect abundances were similar between dry and wet sides of the island of Hawai’i (t = 0.66, df = 54, p = 0.51). In contrast, counts using the same method in the Australian tropics averaged 44.5 insects around each light (SE = 5.5, range 24 to 64, N = 15) in the dry season (May to October) and 157.4 (SE = 5.14, range 85 to 240, N = 17) in the wet season (November to April).

### Distance between successive diurnal retreat-sites

**Fig 2:**
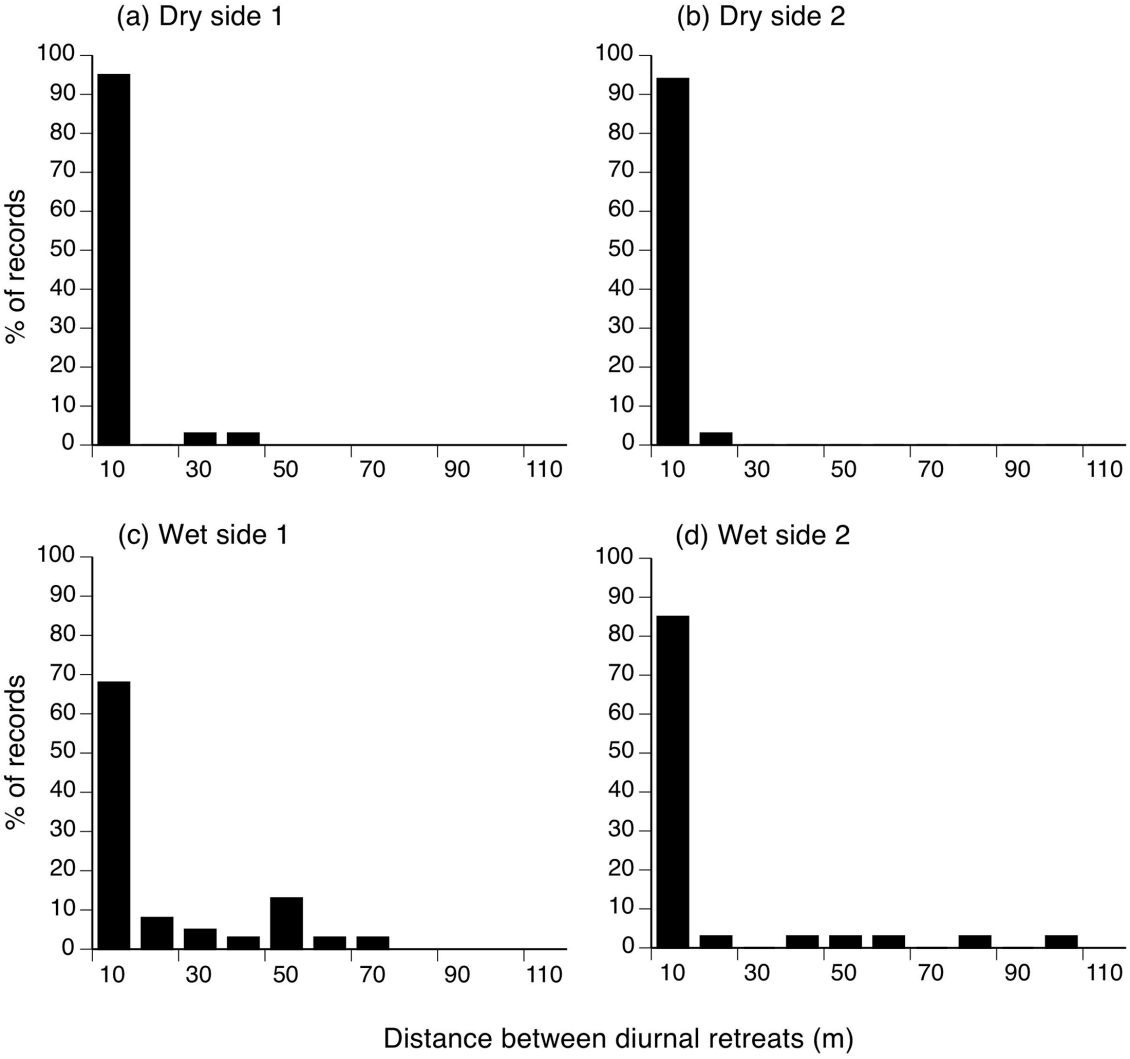
Distances moved by toads between diurnal retreats. Figure shows movement distances between successive diurnal shelters made by 40 radio-tracked cane toads on the island of Hawai’i. Data are shown for two sites on the leeward (dry) side of the island, and for two sites on the windward (wet) side.

Three toads moved distances of >100 m overnight (140 m, 140 m, 520 m; all from Dry side 2) but all others selected diurnal retreats that were close to the one used the previous night (Fig 2). The mean distance between successive retreat-sites was 11.1 m (SE = 3.67) if all records were included, and 7.01 m (SE = 1.5) if the three atypically large movements were excluded. In 70% of cases (111 of 158), the toad was found in the same shelter as the previous day. Frequently, multiple toads used the same shelter-site at the same time. The three long-range movements show that toads are capable of dispersing across this harsh landscape. The longest move (520 m) was by a male toad (98 mm SUL, 119 g) on the night it was released. It dispersed across a wide lava flow between greens on the golf course into a small crevice at one edge of the lava flow, and was found in that same crevice every day thereafter for the duration of radio-tracking.

### Nocturnal movements

Spool-tracking showed that toads left their shelter-sites every night, even if they were found in the same diurnal retreat on successive days. Net displacement (furthest straight-line distance from retreat site) per night during spool-tracking averaged 27.62 m (SE = 10.36; range 1 to 300 m) whereas the mean distance travelled per night was 115.91 m (SE = 15.78; range 5 to 360 m).

**Fig 3:**
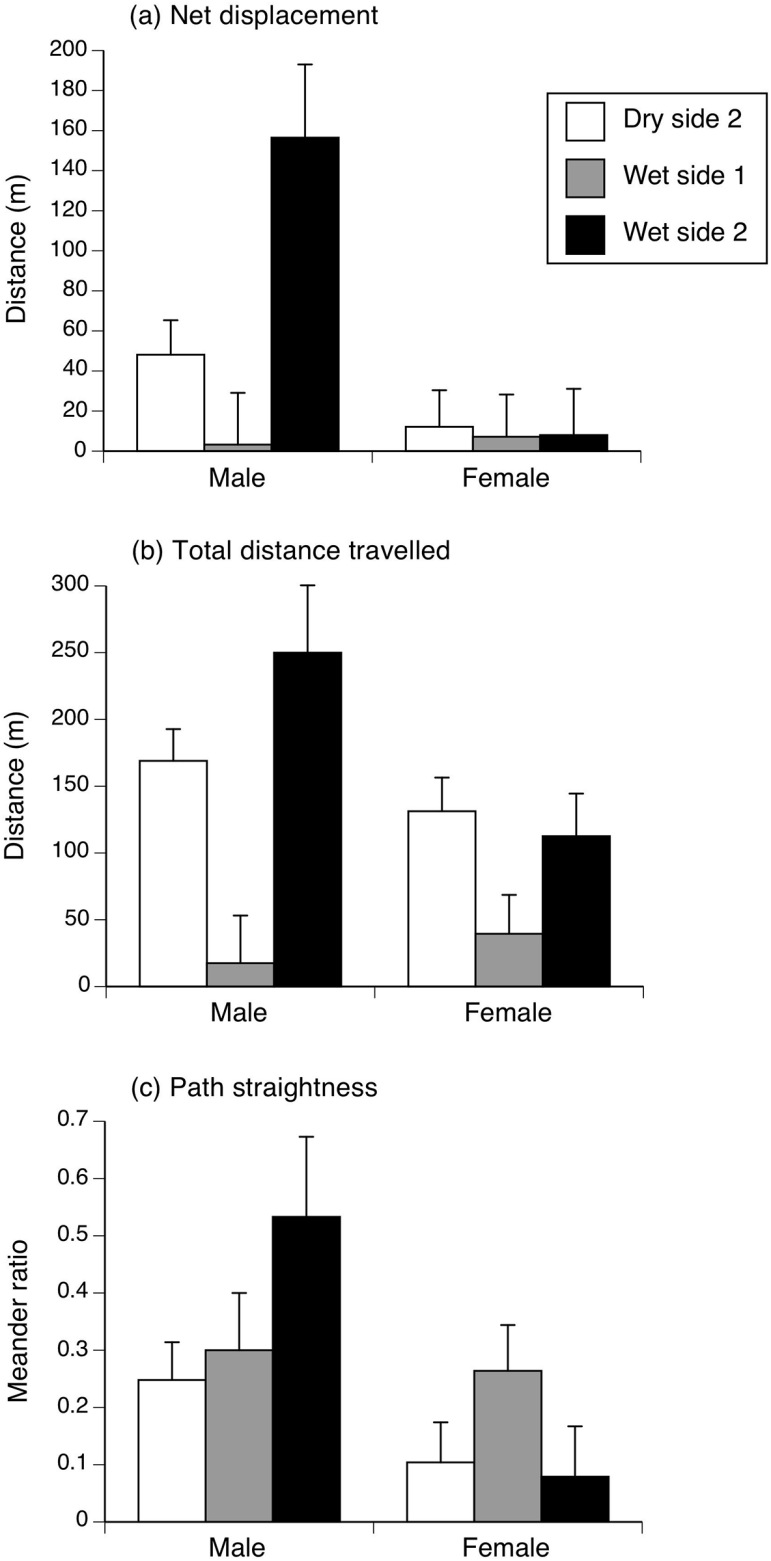
Nocturnal movements of toads. Figure shows movement patterns of 39 spool-tracked male and female cane toads at three sites on Hawai’i: (a) net displacement between successive diurnal retreat-sites, (b) total distance travelled, and (c) path straightness (net displacement as % of total distance travelled). Panels show mean values and associated standard errors.

ANOVA with sex and site as factors showed that net displacements differed between sexes (F_1,33_ = 9.04, p < 0.01) and sites (F_2,33_ = 4.01, p < 0.03), with males travelling further than females at one dry-side site but not the other (Fig 3a; interaction F_2,33_ = 4.01, p < 0.03). Total distances travelled also varied among sites (F_2,33_ = 11.52, p < 0.001) but were not significantly affected by sex (F_1,33_ = 3.41, p = 0.08; interaction sex*site, F_2,33_ = 2.24, p = 0.13; Fig 3b). As a consequence of these two patterns, path straightness was greater in males than females (net displacement was 36% vs. 15% of total movement, respectively; F_1,33_ = 7.67, p < 0.01) but did not differ among sites (F_2,33_ = 1.40, p = 0.26; interaction, F_2,33_ = 2.07, p = 0.15; see Fig 3c).

### Habitat use

**Fig 4:**
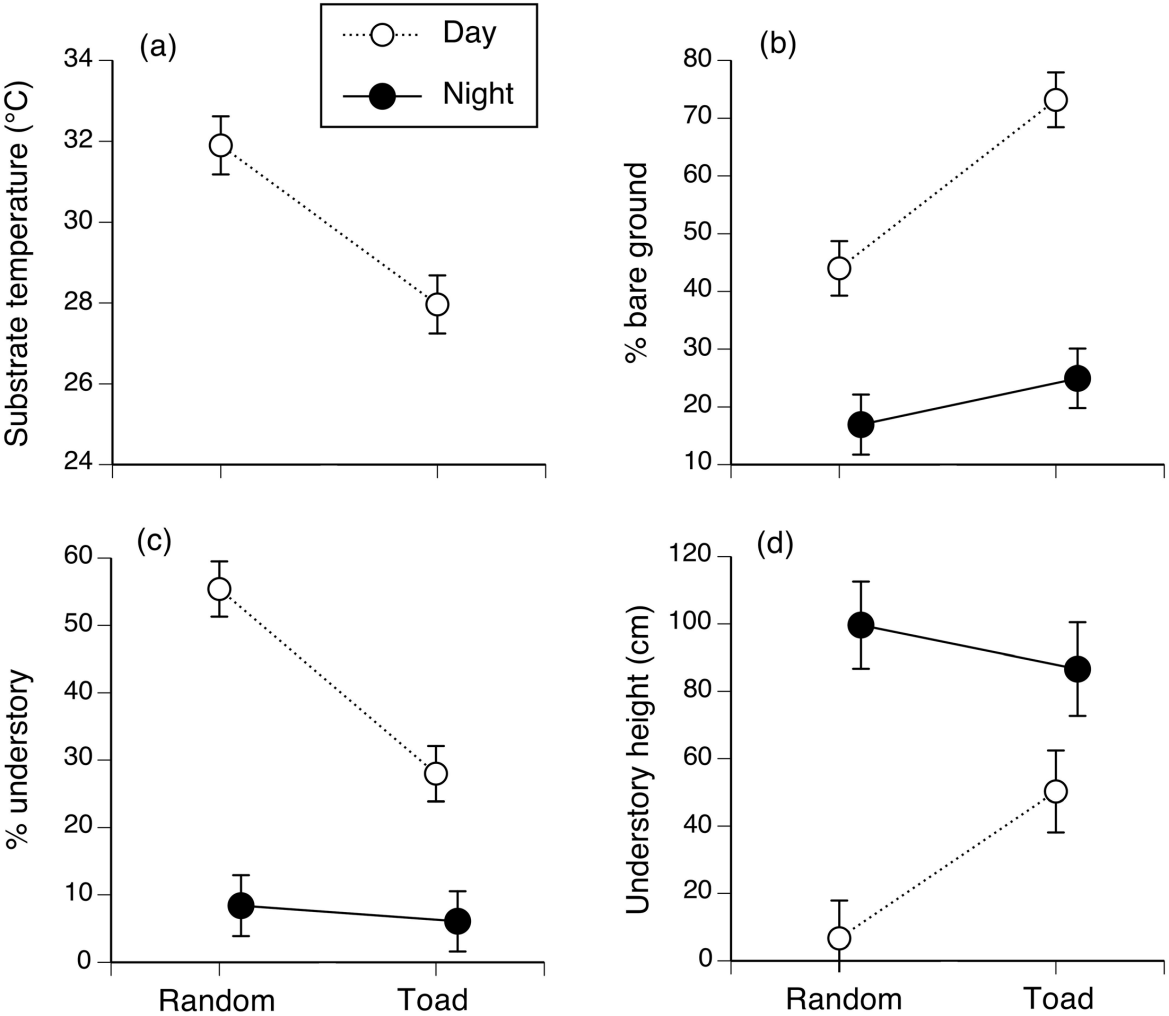
Microhabitat selection by toads on Hawai’i. Figure shows microhabitat use of 40 radio-tracked and spool-tracked cane toads on the island of Hawai’i. The panels show interactions between time (day vs. night) and toad habitat selection (used places vs. nearby available sites that were not used): (a) substrate temperature, as recorded using an infrared thermometer (recorded only by day); (b) the % of bare ground within a 1-m quadrat surrounding the toad, compared to a randomly-chosen site 5 m away; (c) the % of understory vegetation within a 1-m quadrat surrounding the toad, compared to a randomly-chosen site 5 m away; and (d) the height of understory vegetation within a 1-m quadrat surrounding the toad, compared to a randomly-chosen site 5 m away. Figure shows mean values and associated standard errors.

Our surveys yielded an extensive data set on attributes of the microhabitats used by toads, as well as nearby available (but unused) microhabitats. Analysis revealed many non-random patterns. For example, toads were active at night in areas with less bare ground, a more open canopy, less dense understory, and less leaf-litter than were found around their diurnal retreat-sites (Table 2, Fig 4). Similarly, conditions at the place a toad was found differed from those at a nearby randomly-selected (unused) site; toads selected diurnal retreat-sites that gave them access to bare ground, and higher and less dense understory (Table 2, Fig 4). Overall, habitat selection by toads was strongly non-random by day, but not by night (Fig 4).

**Table 2:** Effects of site, sex, time (day vs. night) and usage by toads (used vs. random nearby site) on attributes of microhabitats used by (and available to) radio-tracked cane toads on the island of Hawai’i.

Habitat parameter	Site DF	Site F	Day-night DF	Day-night F	Toad sex DF	Toad sex F	Toad vs. Random DF	Toad vs. Random F	DN*TR DF	DN*TR F
% bare ground	3,35.1	0.45	1,79.0	33.51***	1,31.0	2.25	1,35.1	32.15***	1,71.3	4.19*
% canopy cover	3,36.6	3.21*	1,31.5	2.55	1,34.3	0.01	1,16.2	2.51	1,44.8	0.09
Canopy height (m)	3,23.7	0.49	1,0.5	3.22	1,20.3	0.40	1,21.2	1.32	1,0.62	2.31
% litter	3,43.2	0.33	1,57.3	8.79**	1,42.5	2.86	1,36.7	0.80	1,73.7	0.71
% understory vegetation	3,35.1	0.59	1,63.4	50.40***	1,32.1	0.003	1,39.3	17.29**	1,66.6	8.38*
Understory height (m)	3,25.9	3.53*	1,41.5	33.2***	1,31.4	0.003	1,28.6	0.15	1,50.8	1.88

The Table shows DF and F-values from ANOVA with site, day-night, toad sex and used vs. available as factors, including toad ID as a random factor. Dependent variables were habitat descriptors. Table shows main effects plus the interaction term DN (= day-night)* TR (= toad-random).

Statistical significance (not corrected for multiple comparisons) is shown as

* p < 0.05;

** p < 0.01;

*** p < 0.001;

all p < 0.05 are in boldface font.

**Fig 5:**
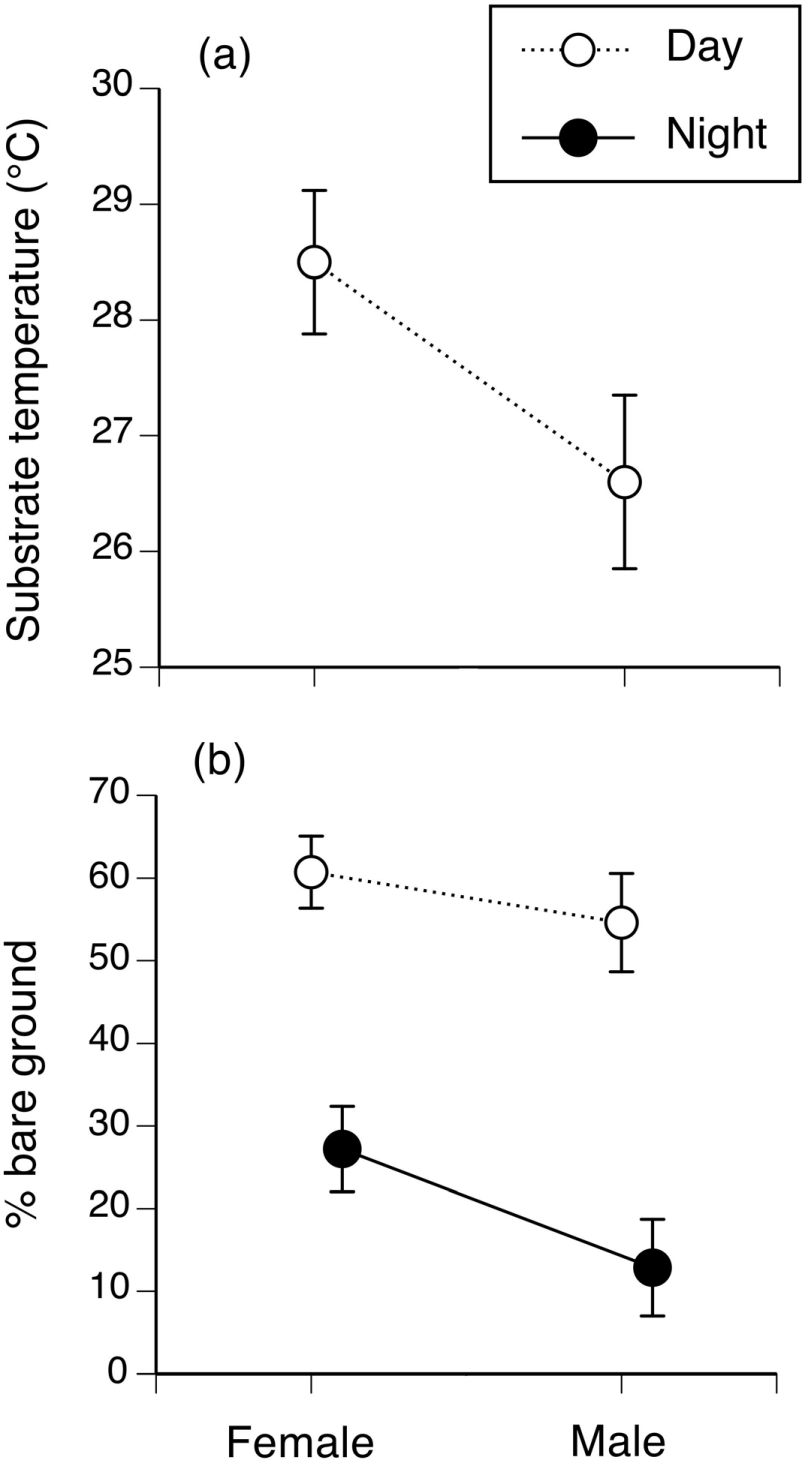
Microhabitat selection as a function of sex. Figure shows microhabitat use of 40 radio-tracked and spool-tracked cane toads on Hawai’i. The panels show interactions between time (day vs. night) and toad sex: (a) substrate temperature, as recorded with an infrared thermometer (recorded only by day); and (b) the % of bare ground within a 1-m quadrat surrounding the toad. Figure shows mean values and associated standard errors.

Interpretation of these patterns is complicated by interactions between time of day, toad sex, and whether or not a site was used by a toad. Nonetheless, most main effects are straightforward because the interactions did not involve reversals of general patterns (Figs 4 and 5). Figs 4 and 5 plot several of these interaction effects. Inspection of the interaction between time (day vs. night) and usage (used vs. available sites) shows that the animals selected areas of relatively bare ground (versus the availability of that habitat type, especially by day: Fig 4b), reflecting their frequent diurnal use of burrows within open areas. Some of those sites were in lava flows, but most radio-tracked toads spent the day in anthropogenically-created retreat-sites, which were cooler than adjacent sites and often, in bare areas shaded by a dense but high understory (Fig 4). A toad’s sex also influenced its habitat use. Female toads were found in areas with more bare ground (Fig 5).

## Discussion

Cane toads were abundant and widely distributed across all three Hawai’ian islands that we surveyed, even in severely arid habitats (Table 1). At first sight, lava flows (which dominate the dry side of the island of Hawai’i) appear as an arid moonscape that is utterly unsuitable for a large anuran. The cane toad’s ability to thrive in Hawai’i is even more surprising given the high and relatively aseasonal rainfall over most of its native range [24]. North of Kailua-Kona on the western side of Hawai’i, the only green patches in the landscape are around freshwater seeps (behind the beaches), suburban gardens, parks and golf courses. The abundance of cane toads in Hawai’i reflects the species’ ability to exploit scattered patches of suitable abiotic and biotic conditions within a harsh matrix.

Thermal conditions at low elevations on Hawai’i are stable, and high enough to support toad activity year-round [19,25]. Predators are scarce, but may include large invertebrates (that can consume eggs, larvae and metamorphs [26]) and the mongoose (capable of consuming adult toads, but probably unlikely to do so [7]). The major challenges facing toads over most of Hawai’i involve food and water. Flying insects are rare (around 1 to 2% of the abundances we recorded in tropical Australia), and our dissections and observations suggest that invasive cockroaches (especially the Surinam cockroach *Pycnoscelus surinamensis* and the American cockroach *Periplaneta americana*) are the primary prey resource for Hawai’ian cane toads. Both of these insect species are most common around human habitation [27]. Water for rehydration and breeding also is rare in undisturbed habitats, especially on the dry side of the island. With a few exceptions, the only permanent freshwater pools suitable for breeding are those created by human activities [19].

Hawai’ian cane toads thus live in oases of plenty amidst an inhospitable world. The small spatial scale of suitable habitat patches favors philopatry (most dispersers will encounter dangerously dry conditions and little food) and reliance upon a small area wherein the critical resources (such as the location of a cool moist shelter site) can be learned [18,28]. Our data accord with this prediction. The toads we tracked spent the day (often in groups) within cool shelter-sites, to which each toad typically returned the following day. They moved extensively during the night, but (especially in females) those movements did not take them far from their diurnal retreats.

We noted several differences between Hawai’ian toads and their conspecifics in Australia. Australian toads frequently use roads as dispersal corridors [29] but we rarely saw toads on the (dry) roads of Hawai’i, even in areas where the animals were abundant on (moist) suburban lawns. Likewise, artificial lights at night attract toads in Australia [7,30], but not in Hawai’i. The virtual absence of flying insects has two consequences: it renders lights irrelevant as foraging sites for toads, and it reduces the distances that insects disperse away from their usual habitats. Thus, prey may be more concentrated in moist habitat patches in Hawai’i than in Australia. Lastly, we noted arboreal foraging in Hawai’ian toads whereas we have not seen this in our extensive work in Australia; the scarcity of flying insects, and abundance of cockroaches on fruit-bearing trees, may explain this behavior of the Hawai’ian toads.

The sex differences in movement patterns and habitat use of Hawai’ian toads mirror trends seen in other populations of this species (and in other anurans [31]). Reproductive males congregate near waterbodies and call to attract females, whereas females spend their time in food-rich areas away from water [32]. Hawai’ian toads feed heavily on the frugivorous cockroach *Pycnoscelus surinamensis*, which attains high abundances on trees and fallen fruit (GW-F, pers. obs.). Several of our spool-tracked toads travelled backwards and forwards beside hedge-rows, presumably feeding on cockroaches that had gathered there. Female toads moved from diurnal shelters to nearby foraging sites, criss-crossed those areas overnight, then returned to their shelters. In contrast, males travelled further, and often via straighter paths, to the nearest breeding pond. Many of the differences in habitat use between male and female toads likely reflect this divergence. For example, female toads may be found in locations with bare ground and understory vegetation because such sites are in drier areas (closer to foraging sites, further from water).

Paradoxically, toads that live in the most arid sites may be the most aquatic. Cane toads survive in arid regions of Australia by remaining in or near permanent waterbodies [33–35]. The same pattern seems to occur on Hawai’i, with toads widely dispersed across the (relatively) mesic landscape of the windward (wet) side of the island, but heavily concentrated in well-watered patches (usually golf courses) on the leeward (dry) side. Wet-side toads inhabit a world where hydric conditions fall along a continuum, whereas dry-side toads experience a stark dichotomy between highly mesic and highly xeric available habitat.

Several radio-tracking studies have been conducted on cane toads in other parts of their range, allowing us to compare the Hawai’ian toads to other populations. Although comparison is weakened by methodological divergences, information from the cane toad’s native range suggests limited vagility. In Panama, Zug and Zug [18] reported home ranges of 160 m^2^ (based on 27 adult animals). In Puerto Rico, Carpenter and Gillingham [36] reported that 14 marked adults returned to the same sites where they were first found, after moving up to 165 m away in the interim. Likewise, Brattstrom [28] (in Panama) and Boland [37] (in Queensland, Australia) reported that toads returned to their capture site even if translocated. In Amazonian Brazil, spool-tracking of 203 animals led Bayliss [38] to conclude that they usually move around 130 m per night, and his recaptures of 670 marked adult toads revealed net displacements of around 30 m per day. By comparison, we recorded mean nocturnal movements of 115 m, and displacements of 11 m. All of these records, then, are similar to the results we found in Hawai’i.

The movement patterns and habitat use of Hawai’ian toads also resemble those of conspecifics from eastern Australia, at least in broad terms [39,40]. In the wet tropics of coastal Queensland where toads were released in 1932, radio-tracking at two sites showed average daily displacements of 17 and 20 m (means calculated from raw data used in the analysis by Alford et al. [41], see Fig 2 of that paper). Mean net daily displacements were 87 and 65 m at the same two sites. That spatial scale is broadly similar to that we have recorded in the current study, except that net displacements were greater, and total movement lower, than we recorded for the Hawai’ian toads. That difference may reflect the scarcity of diurnal retreat sites in Hawai’i (encouraging re-use of the previous site) combined with lower prey densities (stimulating more extensive foraging movements).

In contrast to these records of relative philopatry in cane toads over most of their geographic distribution, radio-tracked toads at the expanding range edge in northwestern Australia travelled an average of 259 m per night during suitable weather conditions, with average nightly displacements of 134 m [41]. Occasional individuals travelled much further, with one toad moving >20 km in 30 nights [42]. In summary, cane toads in most populations (including Hawai’i) are relatively sedentary; but invasion-front animals from western Australia are highly vagile. This pattern is consistent with the hypothesis that the high dispersal rates of invasion-front toads in Australia are an evolved response to sustained range expansion [14,15,43,44].

The high mean distances travelled by spool-tracked Hawai’ian toads (>150 m per night), despite their low net displacements, emphasize the great mobility of these animals. Anurans of most species rarely travel so far on routine foraging trips [31]. The cane toad’s facility for long-distance movements also is exemplified by the occasional long-distance dispersal events across harsh terrain, notably the male that travelled >500 m in a night across a lava flow. The ability to traverse long stretches of arid landscape enabled cane toads to spread out from irrigated sugar-cane plantations to other moist microenvironments even on the leeward (dry) sides of the Hawai’ian islands, and thus, to persist after the sugar-cane industry collapsed in this area a few decades later [11].

In combination with research from other parts of the species’ native and invaded range, the Hawai’ian data clarify reasons for the spectacular global success of *Rhinella marina*. Individual cane toads flexibly adjust their activity patterns and habitat use to temporal and spatial variation in the availability of food and water [21,32] and a capacity for rapid evolutionary change fine-tunes toad biology to local challenges [16]. The large body size of adult cane toads confers high mobility, allowing them to traverse wide swathes of unsuitable habitat to locate scattered patches that provide better resources. In addition, large body size may prolong survival times under desiccating conditions, and enhance tolerance of the polluted water often found around human habitations [45]. More broadly, the key to the success of *Rhinella marina* as an invader may lie in its ability to exploit resource-rich patches in a habitat that is spatially and temporally heterogeneous, by moving long distances (if needed) through an inhospitable matrix. Although manifested only occasionally in the native range and in other invaded sites such as Hawai’i, that capacity preadapted cane toads to spreading across thousands of kilometers in Australia.

## Supporting Information

S1 Table The data underlying this study has been made available in a table of supporting information. (XLS)
